# Supplementary material for: A new preferentially outcrossing monoicous species of Volvox sect. Volvox (Chlorophyta) from Thailand
Source: PLoS One. 2020 Jul 2;15(7):e0235622. doi: 10.1371/journal.pone.0235622 (PMC7332039; doi:10.1371/journal.pone.0235622)
Supplement: S1 Fig — (DOCX) [file pone.0235622.s001.docx]

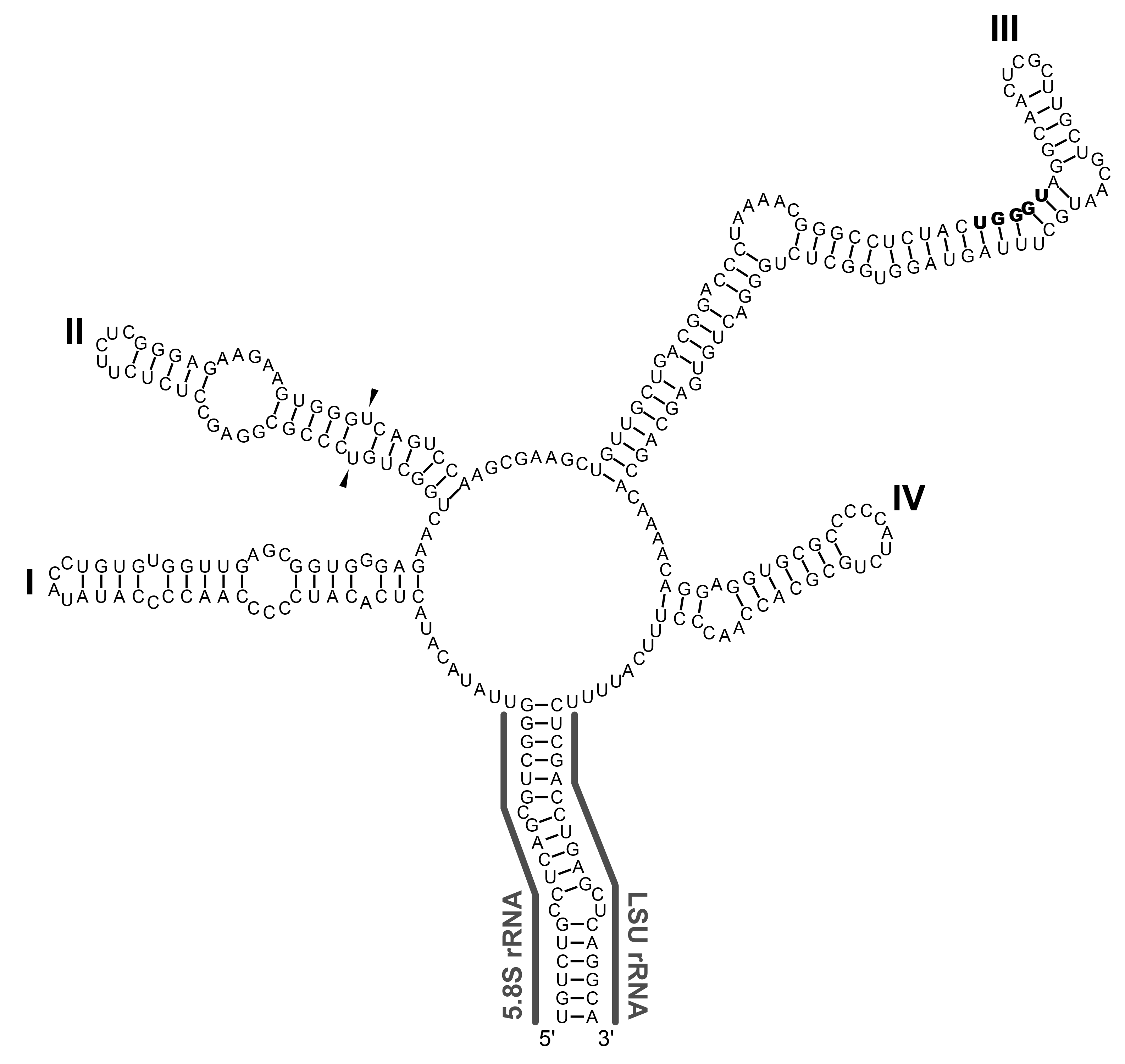


**S1 Fig. The secondary structure of nuclear ribosomal DNA (rDNA) internal transcribed spacer 2 (ITS-2) transcript of five strains of *Volvox longispiniferus* sp. nov., including the 3’ end of the 5.8S ribosomal RNA (rRNA) and the 5’ end of the large subunit of rRNA (LSU rRNA).** Secondary structure of nuclear rDNA ITS-2 was drawn using VARNA version 3.9. Note the U-U mismatch in helix II (arrowheads) and the YGGY motif (UGGGU) on the 5’ side near the apex of helix III (boldface), common structural hallmarks of eukaryotic nuclear rDNA ITS-2 secondary structures. Note that all five strains of *V. longispiniferus* (Table 1) have the identical ITS-2 sequences.
