# Supplementary material for: A new preferentially outcrossing monoicous species of Volvox sect. Volvox (Chlorophyta) from Thailand
Source: PLoS One. 2020 Jul 2;15(7):e0235622. doi: 10.1371/journal.pone.0235622 (PMC7332039; doi:10.1371/journal.pone.0235622)
Supplement: S2 Fig — (DOCX) [file pone.0235622.s002.docx]

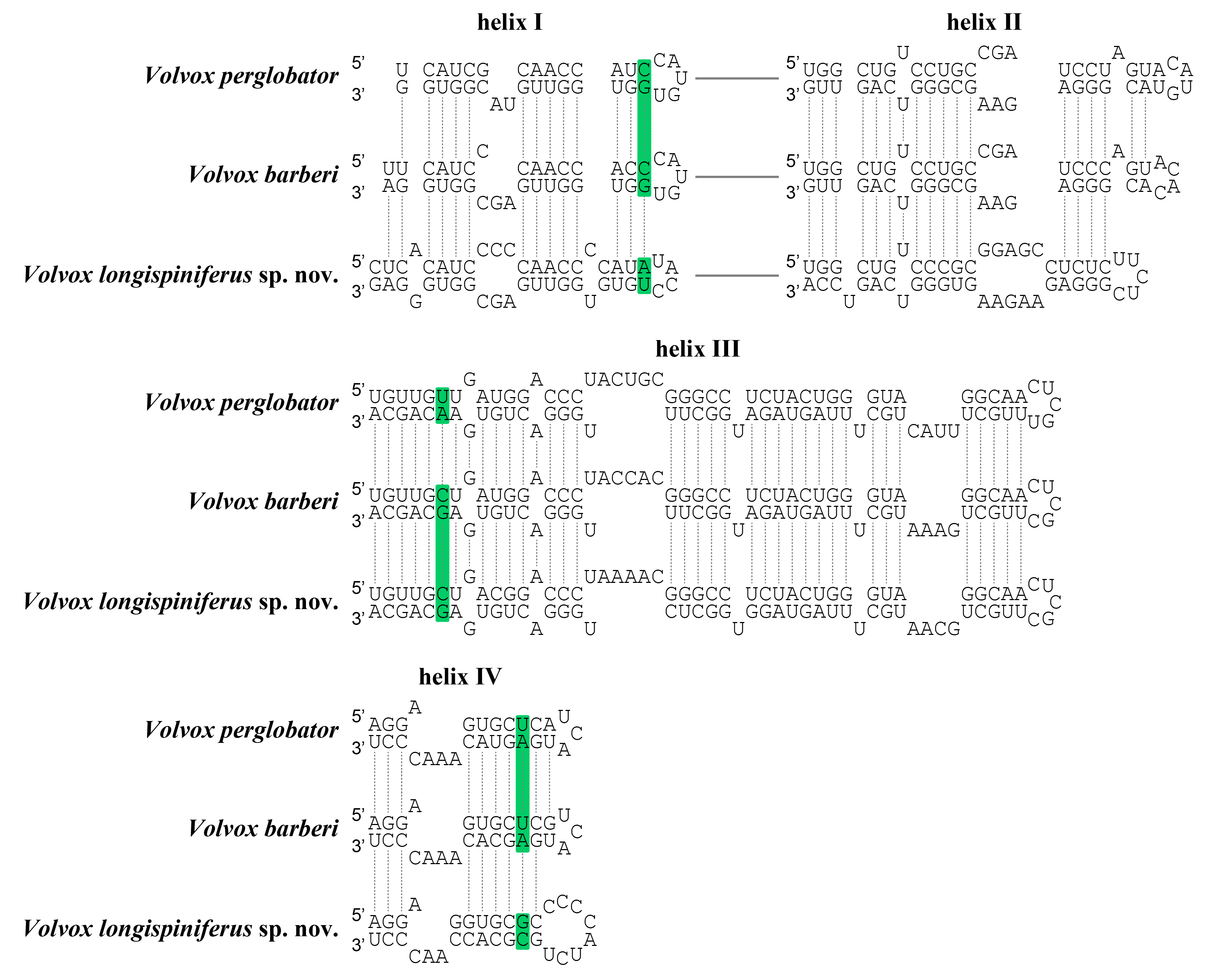


**S2 Fig. Comparison of helices of the secondary structure of nuclear ribosomal DNA internal transcribed spacer 2 transcripts between *Volvox longispiniferus* sp. nov. and its related strain/species (Figs 4–6).** Green backgrounds indicate compensatory base changes between *V. longispiniferus* and the other species.
