# Supplementary material for: A new preferentially outcrossing monoicous species of Volvox sect. Volvox (Chlorophyta) from Thailand
Source: PLoS One. 2020 Jul 2;15(7):e0235622. doi: 10.1371/journal.pone.0235622 (PMC7332039; doi:10.1371/journal.pone.0235622)
Supplement: S1 Table — (DOCX) [file pone.0235622.s003.docx]

**S1 Table. Results of zygote formation in eggs per sexual spheroid from three types of sexually induced cultures of *Volvox longispiniferus* sp. nov.**

| Number of zygotes formed (Nz) | Number of eggs ^a^ (Ne) | Rate of zygote formation (Nz/Ne) ^b^ | Type of sexual culture ^c^ | Strain(s) |
| --- | --- | --- | --- | --- |
| 4 | 28 | 0.142857143 | 1 | 1101-NZ-4 x 5 |
| 2 | 32 | 0.0625 | 1 | 1101-NZ-4 x 5 |
| 10 | 47 | 0.212765957 | 1 | 1101-NZ-4 x 5 |
| 6 | 31 | 0.193548387 | 1 | 1101-NZ-4 x 5 |
| 6 | 28 | 0.214285714 | 1 | 1101-NZ-4 x 5 |
| 8 | 39 | 0.205128205 | 1 | 1101-NZ-4 x 5 |
| 8 | 43 | 0.186046512 | 1 | 1101-NZ-4 x 5 |
| 8 | 58 | 0.137931034 | 1 | 1101-NZ-4 x 5 |
| 8 | 32 | 0.25 | 1 | 1101-NZ-4 x 5 |
| 6 | 43 | 0.139534884 | 1 | 1101-NZ-4 x 5 |
|  |  | 0.174459784 (average) | 1 |  |
| 8 | 43 | 0.186046512 | 2 | 1101-NZ-4 |
| 0 | 53 | 0 | 2 | 1101-NZ-4 |
| 7 | 55 | 0.127272727 | 2 | 1101-NZ-4 |
| 10 | 51 | 0.196078431 | 2 | 1101-NZ-4 |
| 4 | 55 | 0.072727273 | 2 | 1101-NZ-4 |
| 6 | 51 | 0.117647059 | 2 | 1101-NZ-4 |
| 9 | 50 | 0.18 | 2 | 1101-NZ-4 |
| 5 | 50 | 0.1 | 2 | 1101-NZ-4 |
| 9 | 50 | 0.18 | 2 | 1101-NZ-4 |
| 9 | 45 | 0.2 | 2 | 1101-NZ-4 |
| 4 | 47 | 0.085106383 | 2 | 1101-NZ-5 |
| 2 | 30 | 0.066666667 | 2 | 1101-NZ-5 |
| 8 | 40 | 0.2 | 2 | 1101-NZ-5 |
| 5 | 40 | 0.125 | 2 | 1101-NZ-5 |
| 1 | 30 | 0.033333333 | 2 | 1101-NZ-5 |
| 6 | 41 | 0.146341463 | 2 | 1101-NZ-5 |
| 2 | 45 | 0.044444444 | 2 | 1101-NZ-5 |
| 3 | 34 | 0.088235294 | 2 | 1101-NZ-5 |
| 4 | 35 | 0.114285714 | 2 | 1101-NZ-5 |
| 4 | 40 | 0.1 | 2 | 1101-NZ-5 |
|  |  | 0.118159265 (average) | 2 |  |
| 8 | 55 | 0.145454545 | 3 | 1101-NZ-4 |
| 8 | 42 | 0.19047619 | 3 | 1101-NZ-4 |
| 2 | 53 | 0.037735849 | 3 | 1101-NZ-4 |
| 7 | 44 | 0.159090909 | 3 | 1101-NZ-4 |
| 3 | 42 | 0.071428571 | 3 | 1101-NZ-5 |
| 3 | 30 | 0.1 | 3 | 1101-NZ-5 |
| 3 | 30 | 0.1 | 3 | 1101-NZ-5 |
| 4 | 30 | 0.133333333 | 3 | 1101-NZ-5 |
| 1 | 39 | 0.025641026 | 3 | 1101-NZ-5 |
|  |  | 0.107017825  (average) | 3 |  |

^a^ Total number of unfertilized eggs and zygotes.

^b^ Data from each of sexual cultures 1–3 do not differ significantly from that which is normally distributed based on the Kolmogorov-Smirnov test of normality in Social Science Statistics <https://www.socscistatistics.com/tests/kolmogorov/default.aspx>. Student’s *t*-test for three pairs of the three data sets shows significant difference [corrected probability values (corrected *p*) < 0.05 (*p* × 3, based on Bonferroni correction)] between sexual cultures 1 and 3 (corrected *p* = 0.047). Corrected *p* between sexual cultures 1 and 3, and between 2 and 3 are 0.052 and 0.955, respectively.

^c^ Sexual culture 1: 1.0 mL cultures including immature sexual spheroids from two different cultures were mixed and inoculated into 10–11 mL fresh USVT medium in Petri dishes (57 × 16 mm); Sexual culture 2: 2.0 mL culture including immature sexual spheroids from a single culture was inoculated into 10–11 mL fresh USVT medium in Petri dishes (57 × 16 mm); and Sexual culture 3: a single immature sexual spheroid was isolated by a micropipette and inoculated into 0.5 mL fresh USVT medium in a tissue culture plate.
